# Spatio-Temporal Distribution Characteristics and Drivers of PM_2.5_ Pollution in Henan Province, Central China, before and during the COVID-19 Epidemic

**DOI:** 10.3390/ijerph20064788

**Published:** 2023-03-08

**Authors:** Pengcheng Lv, Haoyu Zhang, Xiaodong Li

**Affiliations:** College of Geography and Remote Sensing Sciences, Xinjiang University, Urumqi 830017, China

**Keywords:** Henan Province, PM_2.5_, spatial and temporal distribution, spatial Durbin model, socioeconomic factors

## Abstract

PM_2.5_ is the main cause of haze pollution, and studying its spatio-temporal distribution and driving factors can provide a scientific basis for prevention and control policies. Therefore, this study uses air quality monitoring information and socioeconomic data before and during the COVID-19 outbreak in 18 prefecture-level cities in Henan Province from 2017 to 2020, using spatial autocorrelation analysis, ArcGIS mapping, and the spatial autocorrelation analysis. ArcGIS mapping and the Durbin model were used to reveal the characteristics of PM_2.5_ pollution in Henan Province in terms of spatial and temporal distribution characteristics and analyze its causes. The results show that: (1) The annual average PM_2.5_ concentration in Henan Province fluctuates, but decreases from 2017 to 2020, and is higher in the north and lower in the south. (2) The PM_2.5_ concentrations in Henan Province in 2017–2020 are positively autocorrelated spatially, with an obvious spatial spillover effect. Areas characterized by a high concentration saw an increase between 2017 and 2019, and a decrease in 2020; values in low-concentration areas remained stable, and the spatial range showed a decreasing trend. (3) The coefficients of socio-economic factors that increased the PM_2.5_ concentration were construction output value > industrial electricity consumption > energy intensity; those with negative effects were: environmental regulation > green space coverage ratio > population density. Lastly, PM_2.5_ concentrations were negatively correlated with precipitation and temperature, and positively correlated with humidity. Traffic and production restrictions during the COVID-19 epidemic also improved air quality.

## 1. Introduction

In recent years, PM_2.5_, the main component of haze, began to enter the public’s view owing to the recognition that haze pollution brings great harm to human health and socio-economic development. As such, many national and international scholars conducted extensive research on PM_2.5_ concentrations using various methods [1,2,3,4,5,6,7,8]. In terms of the scope and content of the study, there is still a lack of analysis of the spatial and temporal evolution patterns of central Chinese provinces on a longer time scale and their relationships with various economic and social factors. Primary focus was mainly on exploring the factors influencing PM_2.5_ formation, such as meteorological conditions and socio-economic factors [9,10,11,12,13,14,15]. For example, Fu et al. [16] studied atmospheric pollutant concentrations and their main drivers in Heilongjiang Province from 2014 to 2018, and showed that temperature, wind speed, air pressure, atmospheric pollution, humidity, and vegetation cover are the primary influencing factors. Jin et al. [17] selected the normalized difference vegetation index (NDVI), precipitation, temperature, wind speed, and elevation data to analyze the effects of each variable on PM_2.5_ in different regions of China. Unfavorable meteorological conditions can cause the rapid accumulation of pollution [18]. Zhong et al. [19] analyzed and estimated the relative contribution of boundary layer meteorological factors (water vapor) to PM_2.5_ pollution growth using surface and vertical meteorological data. Liu et al. [20] investigated the effects of precipitation and wind on PM_2.5_ and PM_10_ concentrations and showed that precipitation has a wet scavenging effect on both. Regarding socio-economic factors affecting PM_2.5_, Zhao et al. [21] found that population density and secondary industry are key to controlling PM_2.5_ pollution. Furthermore, Ji and Yao [22] showed that income, urbanization, and the service sector have significant effects on PM_2.5_ pollution. Jiang et al. [23] quantified the effect of different socio-economic factors, including city size, industrial activities, and residential activities, on PM_2.5_ pollution in Chinese cities and showed that industrial activities contribute more than other factors. City size and residential activities also have significant effects on PM_2.5_ [23]. Several scholars monitored air quality before and during the COVID-19 epidemic based on different national [24,25,26], urban cluster [27,28,29], and large city [2,30,31,32] scales, and found that air quality improved considerably in areas greatly affected by the epidemic [33,34,35]. This indicates that a series of measures, such as social isolation, traffic control, and shutdown of production, which were put in place in the context of the COVID-19 epidemic, also influenced PM_2.5_ production. Sudden interruptions in human activities, which are very rare, provide a unique perspective from which to explore the issue of air pollution management. Henan Province, an agricultural province with a large population located in central China, experienced rapid industrialization and urbanization in recent years, and thus, faces the problem of reconciling rapid economic development with ecological environmental protection. It is located in the north–south transition zone of China’s natural geography, and its ambient air quality is influenced by internal and external natural and social environments. This study, using Henan Province as its survey area, carefully distinguished the relationships between different meteorological and socio-economic factors and PM_2.5_ concentrations. The results contribute to the current knowledge of the effects of meteorological and socio-economic factors and PM_2.5_ in Henan Province, are indicative of the current management of the atmospheric environment in Henan Province, and provide directions for future countermeasures and policies.

## 2. Materials and Methods

### 2.1. Overview of the Study Area

**Figure 1:**
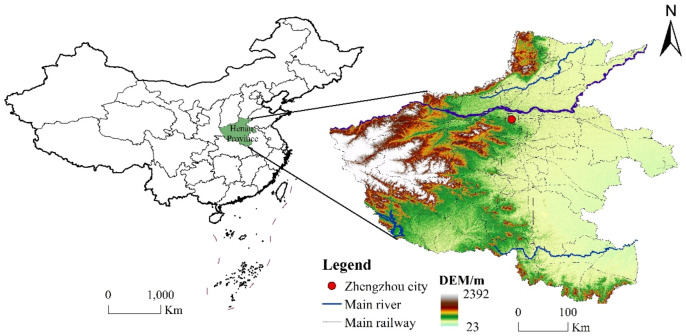
Location and topography of Henan Province.

Henan Province (31°23′–36°22′ N, 110°21′–116°39′ E) is located in the central-eastern part of China, in the middle and lower reaches of the Yellow River, and is one of the most important provinces in this region. The total area of the province is 167,000 km^2^, accounting for 1.73% of the total area of the country. Henan Province borders Anhui Province and Shandong Province to the east, Hebei Province and Shanxi Province to the north, and Shaanxi Province to the west. It has a high topography in the west and a low one in the east, with mountains distributed along the provincial boundary in a semi-circular manner in the north, west, and south, and plain basins in the east-central and southwestern parts (Figure 1). Most of Henan Province is located in a warm, temperate climate zone, spanning the four major basins of the Yangtze River, Huaihe River, Yellow River, and Haihe River, with a vast area of arable land and rich flora, fauna, and mineral resources, making it the main agricultural, and an important mineral, resource province in China.

The study period was 2017–2020, and the study area included 17 prefecture-level cities and 1 province-administered county-level city. In recent years, Henan Province experienced rapid socio-economic development, with its resident population reaching 99.41 million in 2020 and its GDP growing to a total of 5,499.707 billion yuan, making it the fifth largest economy in the country and the first among the central and western provinces, with increasing economic development and living standards. With a well-working transportation system, large population, and dense human production and life, Henan Province is an important industrial and economic agglomeration in China and one of the core pivoting points promoting the rapid development of the western regions and enhancing the comprehensive strength of the country.

### 2.2. Research Data Sources and Processing

Considering the accessibility of the data, GDP, population density, green space coverage, construction output, industrial electricity consumption, number of private cars, energy intensity, and urbanization rate were selected as socio-economic factors affecting the PM_2.5_ concentration; average precipitation, annual average temperature, and humidity were selected as meteorological factors. Impact factor data from 2017 to 2020 were obtained from the *Henan Statistical Yearbook* of each city, the Henan Environmental Status Bulletin, etc. Some of the data were obtained from the resource and environment data cloud platform of the Resource and Environment Science Data Center of the Chinese Academy of Sciences and the National Earth System Science Data Sharing Service Platform, and the maps were produced on the basis of the “Henan Province Map” of the National Geographic Information Public Service Platform. Missing data were supplemented using the mean replacement method. Information on confirmed COVID-19 cases was collected from government bulletins and the news. The results of the multiple covariance analysis of each factor using SPSS v.25.0 show that the variance inflation factor (VIF) of the urbanization rate was 22, which indicates that it may have multiple covariances with population density; therefore, it was removed from the model analysis and the remaining 12 variables were retained.

PM_2.5_ data were hourly pollutant monitoring data from the air quality monitoring stations of the National Environmental Monitoring Center of China, and a total of 18 stations were included in the study area (http://www.weather.com.cn/air/, accessed on 18 November 2022).

### 2.3. Research Methodology

#### 2.3.1. Spatial Correlation Test

In this study, the Moran’s I index was used to measure the global and local spatial autocorrelation of PM_2.5_ in Henan Province [36,37,38], according to which the global spatial autocorrelation can be expressed using Equation (1):

(1) $$I=\frac{k\sum_{i=1}^{k}\sum_{j=1}^{k}W_{ij}\left(x_{i}-\bar{x}\right)\left(x_{j}-\bar{x}\right)}{\sum_{i=1}^{k}\sum_{j=1}^{k}W_{ij}{\left(x_{i}-\bar{x}\right)}^{2}}$$

where k is the number of samples; $x_{i}\;$and$\;x_{j}\;$are the observed PM_2.5_ concentrations in city i and city j, respectively; x is the mean of the observed PM_2.5_ concentrations in all cities in the study area; and $W_{ij}$ is the spatial weight matrix. I values range from −1 to 1, with those greater than 0 indicating that the observations are positively correlated within the region, less than 0 indicating a negative correlation, and 0 indicating no spatial correlation. The local spatial autocorrelation was calculated using Equation (2):

(2) $$I=\frac{\left(x_{i}-\bar{x}\right)}{\frac{1}{k}\sum_{i}{\left(x_{i}-\bar{x}\right)}^{2}}\sum_{j}W_{ij}\left(x_{j}-\bar{x}\right)$$

where an I value greater than 0 indicates a high value being clustered with another high value or a low value clustered with another low one, and positive spatial correlation. If I is less than 0, a high value is adjacent to a low value or a low value is close to a high value, and the spatial correlation is negative.

#### 2.3.2. Spatial Durbin Model

Regional environmental pollution is not only influenced by socio-economic development, but also by the environment of the surrounding area. Thus, the spatial Durbin model, outlined in Equation (3), was chosen to analyze the influence of various factors on PM_2.5_ [38,39]:

(3) I=ρWY+βX+WXθ+ε

where X and Y are the independent and dependent variables, respectively; ρ denotes the endogenous interaction effect coefficient whose numerical magnitude reflects the degree of spatial diffusion or spatial spillover of changes in the dependent variable in other regions; β denotes the regression coefficient; W denotes the spatial weight matrix; θ denotes the exogenous interaction effect coefficient whose numerical magnitude reflects the strength of the interaction of the independent variables; and ε denotes the error. The introduction of a spatial weight matrix is one of the main differences between traditional and spatial econometric models, and it is necessary to quantify the location of spatial units according to “distance” in model calculations. In this study, the neighboring distance was used, and all geographic units are listed in a two-dimensional table with 0 or 1, where “1” denotes spatially adjacent and “0” reflects spatially nonadjacent. For a system with n spatial cells, the spatial matrix W is expressed as an *n* × *n* binary matrix with symmetry, diagonal elements of 0, and adjacent elements of 1.

## 3. Results and Evolution Characteristics Analysis

### 3.1. Spatial Autocorrelation Analysis

The Moran’s I index of PM_2.5_ concentration values in Henan Province from 2015 to 2018 was analyzed using ArcGIS v.10.8 and resulted in a significance value of less than 0.01, indicating that the distribution of PM_2.5_ concentration values in each region had a positive spatial correlation. Notably, the global Moran’s I index can only reflect the overall clustering characteristics of PM_2.5_ pollution within Henan Province but cannot show the variability among cities and towns. Therefore, considering the heterogeneity among the regions in Henan Province, this study used the local Moran scatter distribution table (Table 1) to further reveal the spatial clustering characteristics of PM_2.5_ concentrations in local areas based on the PM_2.5_ data of each city in 2017, 2018, 2019, and 2020.

The number of cities with Moran scattering points falling in the first and third quadrants of the comprehensive carrying capacity of cities in the Yellow River basin in 2017, 2018, 2019, and 2020 accounted for 38.235% and 29.41% of the total sample, with “High–High” and “Low–Low” being the main types of spatial correlation of the PM_2.5_ concentrations throughout Henan Province and showing the characteristics of the cluster distribution. Specifically, the cities in the first quadrant (High–High) were the provincial capital Zhengzhou City and economically strong cities in the northern region of Henan Province, such as Zhengzhou, Xinxiang City, Puyang City, Jiaozuo City, Kaifeng City, Shangqiu City, Xuchang City, and Shangqiu City. These cities had higher PM_2.5_ pollution levels, showed stronger spatial correlation, and had more apparent spatial spillover effects on other surrounding cities. In contrast, the cities in the second quadrant (Low–High), Hebi, Zhoukou, Sanmenxia, and Pingdingshan, had low PM_2.5_ concentrations. Similarly, the cities in the third quadrant (Low–Low), Luohe, Sanmenxia, Zhumadian, and Xinyang, had lower overall PM_2.5_ pollution levels due to the constraints of each city’s economic, transportation, and resource conditions. Finally, the cities in the fourth quadrant (High–Low) were smaller ones, namely Pingdingshan City, Nanyang City, and Xuchang City. Their spatial driving effect on other cities was weak, and the spatial spillover effect was not evident. Therefore, areas with high PM_2.5_ concentrations should weaken their own Matthew effect, and governments should increase regulations and controls to reduce the PM_2.5_ concentration.

### 3.2. Temporal Pattern Analysis

**Figure 2:**
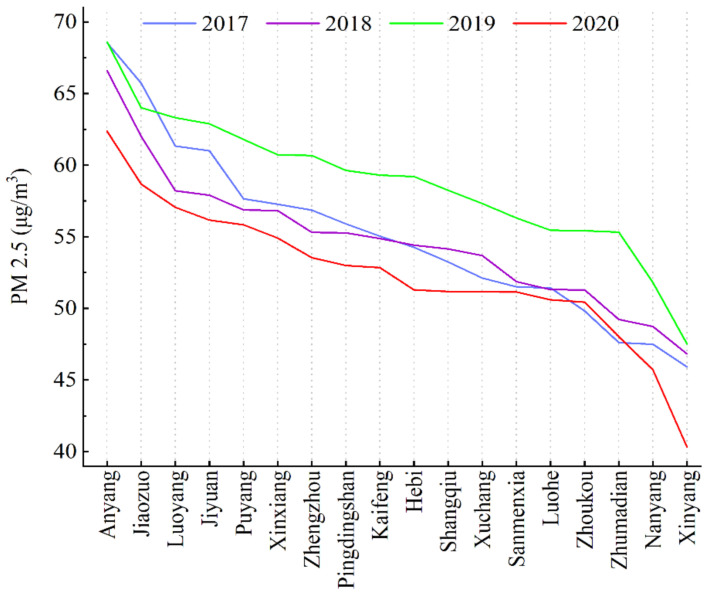
PM_2.5_ concentration distribution trends in various cities of Henan Province.

The results in Figure 2 show that the overall annual average PM_2.5_ concentrations in cities in the study area from 2017 to 2020 showed fluctuating but decreasing trends from 55.153 μg/m^3^ to 52.460 μg/m^3^, and that the air quality was bad. The areas with high PM_2.5_ concentrations mainly included Anyang City, Jiaozuo City, Puyang City, Jiyuan City, Luoyang City, and Kaifeng City, with the annual average PM_2.5_ concentration in Anyang City maintaining the highest value throughout the study period and that in Xinyang City the lowest. In 2017, the PM_2.5_ concentrations were highest in Anyang, Jiaozuo, Luoyang, Jiyuan, Puyang, Xinxiang, Zhengzhou, Pingdingshan, and Kaifeng, reaching or exceeding 55 μg/m^3^, followed by those in Hebi, Shangqiu, Xuchang, Sanmenxia, and Luohe, which remained between 50 and 55 μg/m^3^; at below 50 μg/m^3^ PM_2.5_ concentrations, Zhoukou, Zhumadian, Nanyang, and Xinyang were the cities with the lowest concentrations in the province.

The distribution of PM_2.5_ concentrations was similar in 2018 and 2019, with high values in Anyang, Jiaozuo, Jiyuan, and their surrounding areas reaching 68 μg/m^3^ or more, and lower PM_2.5_ concentrations in Zhumadian, Nanyang, and Xinyang; the distribution of PM_2.5_ in other areas of Henan Province was more evenly spaced, ranging from 50 to 60 μg/m^3^.

From a geographical perspective, the northwest of Henan Province, which included Anyang, Jiaozuo, Luoyang, and Jiyuan City, had high concentrations, indicating serious PM_2.5_ pollution; in comparison, the southeast of the province had lower concentrations. From 2017 to 2019, the average PM_2.5_ concentration increased, while it began to decline in 2020, the inflection point of PM_2.5_, after reaching its maximum value.

### 3.3. Regional Spatial Distributions of Heavy Pollution

**Figure 3:**
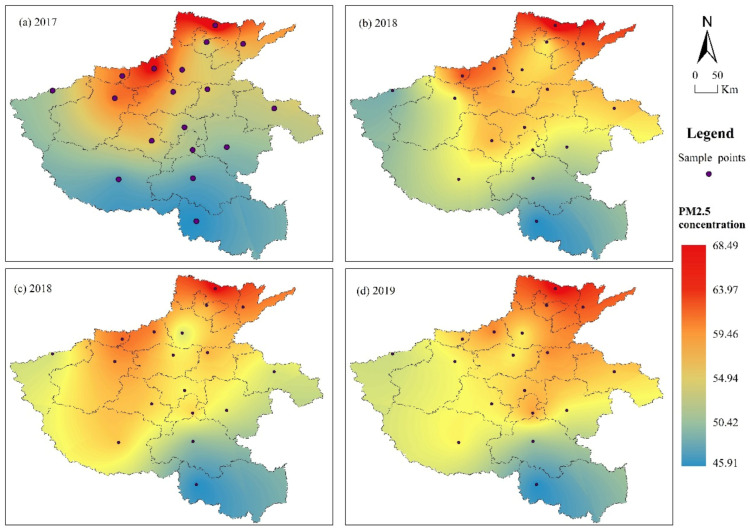
Spatial patterns of PM_2.5_ concentrations and their changes in Henan Province.

The spatial distribution of PM_2.5_ concentrations in urban areas of Henan Province by year was obtained through spatial analysis of the annual average PM_2.5_ concentrations in the 18 cities using the kriging interpolation method of ArcGIS v.10.8. Figure 3 shows that, in 2017, PM_2.5_ pollution was more serious in the northern region, which included Anyang City, Jiaozuo City, Luoyang City, Jiyuan City, Puyang City, and Xinxiang City, while other cities were less polluted. The center with the highest maximum value was located in the region of Anyang City, which had the highest concentration value. In 2018, the span of the high-value area slightly narrowed, highlighting Anyang City, Jiyuan City, Jiaozuo City, Puyang City, Pingdingshan City, and Kaifeng City as areas with high concentrations. Furthermore, even though the pollution in Shangqiu City increased, Anyang City continued to be the city with the highest PM_2.5_ concentration. In 2019, the overall regional pollution levels significantly increased: Anyang City, Jiyuan City, Puyang City, Jiaozuo City, Luoyang City, and Kaifeng City were high-pollution areas, and while the concentration in Luohe City increased, Anyang City remained the area with the highest annual heavy pollution. Notably, some areas saw a slight rebound despite being areas of heavy PM_2.5_ pollution. Finally, in 2020, the overall regional pollution levels declined significantly, but Anyang City, Puyang City, Hebi City, Jiaozuo City, Kaifeng City, and Luohe City remained highly polluted areas.

Comparing the regional heavy PM_2.5_ pollution in 2020 with that in 2019, the following characteristics become apparent: First, the overall value of heavy PM_2.5_ pollution in the region was lower, atmospheric environment quality improved significantly, and the regional distribution of heavy PM_2.5_ pollution showed a relatively uniform trend. Second, the scope of pollution shifted northward, from the southwest region to the north of the traditional high-pollution region, reflecting heavy pollution in the northern regions, which remain the focus of air pollution prevention and control to date.

Overall, the spatial distribution of the PM_2.5_ concentration indicated high values in the north and west and low values in the south and east, with Anyang City, Puyang City, Hebi City, Jiaozuo City, and Luoyang City taken as center points. The most serious pollution in Anyang City, Puyang City, Hebi City, Jiaozuo City, and Luoyang City can be explained by the high proportion of traditional secondary industries in the area, which increases the PM_2.5_ concentration and causes significant air pollution. Among them, the annual average PM_2.5_ concentration in Anyang City exceeded 60 μg/m^3.^ In turn, the concentration distribution in cities in the east and south of Henan Province was more balanced; the southernmost city, Xinyang City, had the lowest distribution of PM_2.5_, and the annual average concentration in Zhengzhou, the provincial capital, decreased continuously.

The PM_2.5_ distribution is greatly related to the topography, climate, and distribution of pollution sources in Henan Province. For example, Zhengzhou City, located in the central region of the province, is characterized by the Taihang Mountains to the north, which are unfavorable to the transportation of air pollutants, causing their accumulation. Similarly, Jiaozuo City, which is closer to the Taihang Mountains, was one of the areas with the highest pollution concentrations. In contrast, Sanmenxia City, the southern area of Luoyang City, and the northwestern area of Nanyang City, had lower PM_2.5_ concentrations owing to their location in mountainous areas with less industrialized, low-emission pollution. Together, the results of the spatio-temporal analysis show that the annual average PM_2.5_ concentrations in cities across Henan Province had a fluctuating but decreasing trend and that the spatial range of high-value pollution areas decreased, which indicates that the air quality in these regions improved.

## 4. Analysis of Influencing Factors

### 4.1. Analysis of the Spatial Durbin Model

Based on the PM_2.5_ data of all cities in Henan Province from 2017 to 2020, the spatial Durbin model analysis was conducted using Stata v.17.0. The regression results are presented in Table 2.

The spatial Durbin model estimation results in Table 2 were analyzed, and, according to the R^2^ and Sigma2 values, the overall fit of the model was good and the reliability of the regression results was high. Thus, an analysis of socio-economic influences was conducted.

#### 4.1.1. Analysis of Socio-Economic Factors

Socio-economic factors, such as the COVID-19 epidemic, energy intensity, industrial electricity consumption, construction output, green space coverage, environmental regulation, and population density, had significant direct effects on the PM_2.5_ concentration, with the coefficients of factors with positive effects being output > industrial electricity consumption > energy intensity, and those of factors with suppressive effects being environmental regulation > green space coverage > population density.

The construction output significantly contributed to PM_2.5_ concentrations owing to smoke and dust emitted at construction sites. Green land coverage had a suppressive effect on atmospheric haze, indicating that vegetation in cities effectively absorbs atmospheric dust and purifies the atmosphere, thus alleviating PM_2.5_ pollution. The coefficients of energy intensity and industrial electricity consumption were positive, indicating that an increase in energy intensity and industrial electricity consumption aggravates haze pollution. High haze results from intense economic development, and energy intensity and industrial electricity consumption are important indicators of economic development. High energy intensity means high energy consumption, and the burning and use of crude oil, raw coal, and other disposable energy sources emit a large amount of exhaust gas, causing serious atmospheric pollution. In turn, industrial electricity consumption reflects the power consumption of cities and is related to regional environmental pollution.

Heavy industries, such as fossil energy, assembly and processing, and machinery manufacturing, account for a high proportion of Henan’s secondary industry, which not only increases ecological and environmental indicators, such as energy consumption, total water consumption, and urban, developed area, but also results in high input and pollution, therefore having a positive impact on PM_2.5_ concentrations. In addition, a large share of industrial electricity consumption corresponds to a more developed industrial sector, the dust generated by the coal power industry is the main source of PM_2.5_, and other generated waste gas particles can contribute to a high PM_2.5_ concentration. It is worth noting that the indirect effects of energy intensity and industrial electricity consumption were also significant, indicating that reducing energy intensity will reduce haze pollution in a region and its adjacent areas, ultimately reducing global haze pollution.

Inconsistent with previous findings, an increase in population density negatively affected the concentration of PM_2.5_, despite a high population agglomeration that increased travel demand and energy consumption. This may be due to the fact that the scale effect of population agglomeration not only increases urban economic development, but also increases the sharing and utilization rates of various facilities and equipment in cities, thus reducing pollution emissions. Additionally, large cities with a high population density tend to be more susceptible to policy tilts, which, together with people’s growing demand for a better quality of life, push urban agglomerations to increase pollution control, and thus, play a role in improving the ecological environment.

The coefficient of the effect of environmental regulation on the PM2.5 concentration was the largest (−23.369 ***), which indicates that environmental regulation significantly inhibits haze pollution. This primarily results from effective environmental regulation policies that promote the transformation of industry, accelerate the construction of the green companies, and reduce the emission of pollutants. After the implementation of environmental regulations, companies generally address pollutants, adopt more environmentally friendly processing materials, strengthen environmental monitoring, and increase the research and development of environmental protection technologies in accordance with the environmental management requirements stipulated by the state. Subject to the influence of pollution resulting from industries, population density, and lifestyle quality, Henan Province is gradually becoming more eco-efficient by coordinating the division of labor in urban industrial chains, balancing the relationship between regional socio-economic development and pollution emissions, highlighting the leading role of core cities with high eco-efficiency, and simultaneously increasing low-carbon transformation, energy conservation, and emission reduction for heavy industries with high energy consumption and pollution.

The overall coefficient for the COVID-19 epidemic was −0.021 ** and it was significantly associated with PM_2.5_. This conclusion is consistent with the findings of Zhu et al. [40], Chauhan and Singh [34], and Zheng et al. [41], who pointed out that policies restricting high-speed traffic and work and production brought about by the outbreak of the COVID-19 epidemic reduced the production of PM_2.5_. The reduction in the emission of pollutants had a spatial synergistic effect, and the ambient air quality in Henan Province improved significantly, which is the main reason for the overall improvement in ambient air quality observed during the survey period, but the basic pattern of PM_2.5_ changes in the region remained unchanged.

Both the GDP and the number of private cars were factors that were not significant in the model: the GDP reflects the degree of economic development of a region, and a high GDP increase generally tends to aggravate the problems of energy consumption and environmental pollution. In turn, the increase in energy consumption leads to a large amount of fuel combustion, exhaust, and sewage. Therefore, economic development generally increases the concentration of PM_2.5_; however, according to the regression results, the coefficient of the PM_2.5_ concentration influenced by the GDP was −0.68, which is inconsistent with expectations. This may be primarily due to the combination of GDP growth and accelerated regional development with the country’s increased efforts to improve environmental protection and management. People’s awareness of environmental issues gradually deepened in recent years, and they are becoming increasingly concerned about environmental pollution. Moreover, with an increase in the population income, the government has sufficient funds available for controlling air pollution, which is evidenced by the implementation of a series of policies to control haze and alleviate haze pollution since 2013. This notion is supported by empirical studies showing that economic development improves haze pollution to some extent.

Interestingly, the increase in the number of private cars led to a decrease in PM_2.5_ concentrations, perhaps due to new technologies replacing older ones, thus reducing energy consumption and lowering the PM_2.5_ pollution. Additionally, the government built a multi-level, diversified modern public transportation system, optimized the layout of railways, vigorously promoted the application of new energy vehicles, and advertised green, low-carbon travel to the public. Lastly, the quarantine policy put in place as a response to the COVID-19 epidemic made residents travel less, and thus, resulted in the negligible impact of the number of private cars on PM_2.5_ pollution.

#### 4.1.2. Analysis of Meteorological Influences

The PM_2.5_ concentrations were negatively correlated with precipitation and air temperature, and the main, direct, and total correlation values of air temperature passed the 0.10 significance threshold, indicating that its suppressive effect was relatively significant. The reason for this may be that the propagation of PM_2.5_ is closely related to atmospheric flow; at higher temperatures, its mobility is greater and airflow is unstable, which is favorable to the propagation of, and a decrease in, PM_2.5_ content. In turn, higher stability of the atmospheric environment is unfavorable for PM_2.5_ propagation. Moreover, the flushing of PM_2.5_ by precipitation leads to a decrease in PM_2.5_ content; however, the precipitation and evaporation of surface moisture in some regions results in higher relative humidity and less precipitation, preventing the spread of PM_2.5_ and causing the accumulation of particulate matter in the atmosphere, thus resulting in an increase in the PM_2.5_ concentration. This relationship between meteorological factors and the PM_2.5_ concentration provides a basis for future air pollution control.

## 5. Suggestions and Discussion

Based on the findings of this study, the following recommendations are suggested to remediate PM_2.5_ pollution in Henan.

(1) Implementation of a differentiated policy for haze control.

The environmental quality problem is more prominent in the northern part of Henan, and because of the differences in resource endowment, economic development level, and the causes of PM_2.5_ in each region, the government should strengthen the governance of cities with more serious haze pollution and increase industrial adjustments; promote the optimization and upgrading of industrial structures and eliminate backward production capacity; strengthen the treatment of key industrial pollution sources; control coal smoke and thermal power plant pollution sources; and strive to reduce the total emissions of particulate matter, sulfur dioxide, and other pollutants.

(2) Adherence to clean energy development strategy.

Actively promote the use of natural gas and other clean energy sources, and vigorously develop and use renewable resources. All regions should continue to reduce coal combustion, increase the use of clean energy, and control total energy consumption to save energy and reduce emissions. Simultaneously, we should increase the supply of new energy sources and promote renewable energy alternatives, such as wind and solar power.

(3) Conduct mutual cooperation and coordination.

Conduct scientific research on haze pollution prevention and control systems in different regions, develop environmental compliance plans, raise awareness of “joint prevention and control”, and enhance the people’s awareness of environmental protection.

(4) Further optimize the industrial structure and promote industrial transformation and upgrading.

Focus on the development of electronic information, light industry, textiles, medicine, and other industries to increase their proportion in the national economy. Additionally, investment in high-tech research and development, development of green industries, promotion of energy conservation, and reduction in emissions should be increased. Moreover, the supervision of environmental protection and reduction in emissions of the three wastes should be strengthened. The control of high energy consumption, high pollution, and high-pollution industries should be increased and industrial restructuring should be vigorously promoted. Thus, on the basis of developing new, low-carbon industries, a shift from traditional high energy-consuming development to a green, low-carbon one will gradually be achieved.

(5) Improvement of the degree of greenery in cities to achieve a haze reduction effect.

Cities should increase capital investment in the construction of green spaces, gradually increase the area of green spaces, and pay more attention to the quality of greenery. Specific attention should be paid to urban green spaces by increasing the late management of and care for greenery and focusing on the coordinated development of urban planning and green space construction, thus improving the impact of vegetation on the environment and the degree of haze pollution.

## 6. Research Outlook

The factors influencing PM_2.5_ concentrations discussed in this paper are not yet fully understood, and other factors related to the concentration of PM_2.5_, such as the correlation between sunshine duration or intensity and urbanization, need to be further investigated.

## 7. Conclusions

In this paper, the following conclusions are drawn from the spatio-temporal distribution characteristics of PM_2.5_ concentrations in Henan Province and the underlying meteorological and socio-economic factors.

(1) Temporally, the overall annual average PM_2.5_ concentrations in Henan Province showed a fluctuating but decreasing trend since the beginning of 2020, with the highest annual average PM_2.5_ concentration value found in Anyang City and the lowest in Xinyang City, which remained consistent throughout the study period.
(2) Spatially, PM_2.5_ concentrations in Henan Province showed positive autocorrelation, with notable fluctuating changes in high concentration areas, which increased from 2017 to 2019, and a consistent high concentration range. During the years in which epidemic control measures were implemented, the PM_2.5_ concentrations decreased significantly, but the basic pattern of air quality did not change. The spatial distribution of PM_2.5_ concentrations weakened from the center of the province to its periphery, with a distribution pattern indicating high values in the north and west and low values in the south and east. The scope of pollution shifted from the southwest region to the traditional high-pollution region in the north, reflecting regional heavy pollution in northern cities, which remains the focus of air pollution prevention and control to date.
(3) The regression analysis based on the spatial Durbin model showed that the socio-economic factors that had a positive effect on PM_2.5_ pollution were construction output value > industrial electricity consumption > energy intensity. In turn, the correlation coefficients of socio-economic factors that had a negative inhibitory effect on the PM_2.5_ concentration were environmental regulation > green space coverage > population density. The absolute value of the regression coefficient of environmental regulation had a negative inhibitory effect on the PM_2.5_ concentration, which differs from the results of other studies. In terms of meteorological influencing factors, PM_2.5_ concentration was negatively correlated with precipitation and temperature and positively correlated with humidity.

## Figures and Tables

**Table 1:** Spatial clustering of PM_2.5_ concentrations in Henan Province, 2017–2020.

Types of Quadrants	High–High	Low–High	Low–Low	High–Low
2017	Puyang, Jiaozuo, Kaifeng, Jiyuan, Shangqiu, Xuchang, Zhengzhou	Hebi, Xinxiang, Zhoukou	Luohe, Luoyang, Nanyang, Sanmenxia, Zhumadian, Xinyang	Pingdingshan, Anyang
2018	Puyang, Jiyuan, Jiaozuo, Kaifeng, Shangqiu, Xuchang, Xinxiang, Zhengzhou	Hebi, Zhoukou	Luohe, Luoyang, Nanyang Sanmenxia, Zhumadian, Xinyang	Pingdingshan, Anyang
2019	Hebi, Jiyuan, Puyang, Anyang, Pingdingshan	Xinxiang, Sanmenxia, Zhengzhou, Shangqiu	Xuchang, Zhoukou, Xinyang, Zhumadian	Jiaozuo, Luoyang, Kaifeng, Luohe, Nanyang
2020	Hebi, Puyang, Anyang, Jiaozuo, Kaifeng, Shangqiu	Xinxiang, Zhengzhou, Zhoukou, Pingdingshan	Luoyang, Nanyang, Sanmenxia, Zhumadian, Xinyang	Jiyuan, Xuchang, Luohe

**Table 2:** Table of the regression results of the spatial Durbin model.

		PM_2.5_		
Variables	Main	Direct	Indirect	Total
COVID-19	−0.023 ***	−0.023 ***	−0.008	−0.021 **
	(−2.93)	(−2.86)	(−0.37)	(−0.19)
Energy intensity	0.011 **	0.011 **	0.024 **	0.812 ***
	(−2.17)	(−2.31)	(−2.02)	(−2.09)
Industrial electricity consumption	0.068 **	0.069 **	0.124 *	0.193 ***
	(−1.76)	(−1.86)	(−1.73)	(−2.03)
GDP	0	0	0	0
	(−0.68)	(−0.63)	(−1)	(−0.57)
Construction output	0.173 ***	0.170 **	−0.171	−0.001
	(−2.76)	(−2.57)	(−0.85)	(−0.00)
Green space coverage	−0.023 ***	−0.025 ***	0.002	−0006
	(−1.81)	(−2.58)	(−0.35)	(−0.012)
Amount of precipitation	−0.007 ***	−0.007 ***	−0.009 **	−0.016 **
	(−3.71)	(−3.80)	(−2.19)	(−3.61)
Number of private cars	0	0	0	0
	(−1.19)	(−1.1)	(−1)	(−0.68)
Temperature	−1.863 *	−1.882 *	2.469	−1.882 *
	(−1.88)	(−1.91)	(−1.13)	(−1.28)
Environmental regulation	−21.023 ***	−22.169 ***	−0.217	−23.369 ***
	(−4.61)	(−4.06)	(−0.09)	(−4.76)
Humidity	0	0	0	0
	(−0.00)	(−0.04)	(−0.11)	(−0.11)
Population density	−0.020 *	−0.019 *	−0.007	−0.019 **
	(−1.81)	(−1.71)	(−0.24)	(−0.37)
sigma2_e				0.185 ***
				(−0.045)
Observations	68	68	68	68
R^2^	0.026	0.026	0.026	0.026
Number of cities	18	18	18	18

Note: ***, **, and * represent significance levels of 0.01, 0.05, and 0.1, respectively; the *t* values are given in parentheses.

## Data Availability

Not applicable.
